# A Suggested Role of Human Growth Hormone in Control of the COVID-19 Pandemic

**DOI:** 10.3389/fendo.2020.569633

**Published:** 2020-11-09

**Authors:** Mohamed Hamdy Elkarow, Amr Hamdy

**Affiliations:** ^1^ Department of General Surgery, Ain Shams University, Cairo, Egypt; ^2^ Faculty of Medicine, Ain Shams University, Cairo, Egypt; ^3^ Department of Obstetrics & Gynecology, Shoubra General Hospital, Ministry of Health and Population, Cairo, Egypt

**Keywords:** coronavirus pandemic, COVID-19, growth hormone, IGF-1, immune system, epidemiology, risk factors

## Abstract

Covid19 is a worldwide pandemic challenge that started in Wuhan, China and spread to almost all countries on the planet within a few months. The causative virus was found to be highly contagious and, until now, considerably difficult to contain. A look at the epidemiological distribution of the disease over the planet has raised a number of questions whose answers could help us understand the behavior of the virus and consequently leads us to possible means of limitation of its spread or even flattening of the curve of morbidity and mortality. After the third decade of life, there is a progressive decline of growth hormone (GH) secretion by approximately 15% for every decade of adult life. The data from highly affected countries suggest a more aggressive course in the elderly, a double-time affection of males more than females, and the vulnerability of some risk groups of patients. Our observation is that GH deficiency is a common factor in all vulnerable patient groups. We think that there is a need for studying the role of growth hormone in the unique epidemiological pattern of Covid-19 so that it might help in the early detection and management of the high-risk groups as appropriate.

## Introduction

Coronavirus disease (Covid-19) is a worldwide pandemic challenge that started in Wuhan, China, in December 2019 and spread to most countries of the world within a few months. The World Health Organization (WHO) declared SARS-CoV-2 as an international public health emergency in January 2020 and as a pandemic in March 2020. Currently, the COVID-19 pandemic has resulted in more than 6 million confirmed cases and more than 370,000 deaths globally. Despite the strict measures adopted by several governments, the disease has imposed an unprecedented burden on health care workers even in countries that have advanced health care services. The socioeconomic impact of the pandemic increased the need for a solution that allows safe easing of restrictive health measures. Primarily, most of the urgent efforts exerted by research centers are directed toward creating a proper vaccine or efficient medical treatment for the virus. However, preventive measures based on understanding the epidemiology of the disease can also be of utmost importance in attenuating its spread.

In this article, we discuss a brief perspective about the possible role of relative growth hormone deficiency (GHD) and/or resistance in the high-risk groups of patients for Covid-19 disease. Our observation is based on the updated epidemiological data of the pandemic and the known levels of growth hormone (GH) in different risk groups of patients as well as the effect of GH on the immune system.

## Why Elderly People Are at a Higher Risk?

Data from the World Health Organization (WHO) reports that the disease affects elderly people much more than young people and children. This pattern is not common in most infectious diseases, which usually affect those age groups who are supposed to be still in the stage of building up their immunity more. For many communicable diseases, young adults and children are at greater risk, for instance, in the largest pandemic in recent history: During the Spanish flu in 1918, children and young adults were at the greatest risk for the pandemic (1).

On the contrary, data from the Chinese Center for Disease Control and Prevention report that children under 19 years of age constitute 2% of the total number (72,314) of Covid-19 cases recorded by February 20, 2020. A very small percentage of those patients aged under 19 years have suffered severe (2.5%) or critical disease (0.2%) (2). On the other hand, severity and case fatality of COVID-19 progressively increased with advancing age. The recorded increase in severity with age is reflected in case reports, in which the mean age was in the range of 50–60 years (3).

There is probably a lacking factor in the elderly that makes them more vulnerable to the disease severity. Growth hormone (GH) level declines in the serum as man advances in age. After the third decade of life, there is a progressive decline of GH secretion by approximately 15% for every decade of adult life. Integrated measurements of daily GH secretion demonstrate that secretion peaks at puberty at about 150 microgram/kg/day and then decreases to approximately 25 microgram/kg/day by age 55 (4). The pediatric age group usually exhibits atypical clinical manifestations of COVID-19, which are mainly milder compared with those of adult patients (5–7), and occurrence of pediatric severe and life-threatening forms are very scarce (8, 9). Children with comorbidities, including chronic kidney and lung disease, diabetes, obesity, sickle cell anemia, malignancy, immune disorders, chromosomal abnormalities, heart disease, and congenital malformations, are more likely to suffer from the severe form of COVID-19 (5–8). Interestingly, infants (less than 12 months) exhibit a more severe form of the disease than other pediatric age groups. GH levels are high in the mid-term fetus and at birth and then decline sharply in the first few weeks and more slowly over the next few months, reaching prepubertal levels by around the age of 6 months (10). The current data suggests that children may get infected but be less symptomatic with less case fatality. The real incidence of infection in children may be unveiled through large screening studies, involving serological tests. The regulation of the GH/IGF-I axis depends on the integrity of the hypothalamus, pituitary, and liver. During aging, the leading factors that contribute to the decline in GH/IGF-I include the changes in signal to the somatotrophs from growth hormone-releasing hormone (GHRH) and somatostatin (SS). Other important factors, such as body composition, exercise, diet, and sleep play a significant role in the age-related decline of the GH level. The phenotypic similarities between aging and adult growth hormone deficiency syndrome combined with this decrease in GH/IGF-I with aging have raised the question of whether aging is a GH deficient state (11). It is worth noting that the reported curve of COVID-19 morbidity and mortality, matches well the pattern of decline of GH level throughout the life of human subjects.

## Why Male Gender Is at a Higher Risk?

Why does the disease affect men at twice the amount it affects women? The emerging trend is that men are much more likely to die of the disease than women. Data from China first revealed a gender gap in death rate with 64% of male patients dying compared to 36% of women, according to the Global Health 50/50 initiative. Figures from Italy, Spain, France, Germany, and the USA, have confirmed the pattern. In the two most affected European countries, 71% of the Covid-19 deaths in Italy were male, and in Spain, almost twice as many men as women have died (12).

The symmetric model in different countries suggests the presence of a relative protective factor in the female gender. It has long been known that GH secretion is greater in women than in men despite similar reference ranges of serum insulin-like growth factor (IGF)-I in adult men and women. It has also been reported that sex steroids influence not only GH secretion but also the local synthesis of IGF-I in target tissues and the expression of the GH receptor in various other tissues (13). The normal range for GH level is typical: for adult males, 0.4 to 10 nanograms per milliliter (ng/mL); for adult females, 1 to 14 ng/mL; and for children, 10 to 50 ng/mL (14). As men grow older, testosterone levels decline gradually with free testosterone concentrations falling by about 50% between ages 25 and 75 years (15). The gradual decline of testosterone with aging is parallel to the age-related fall in growth hormone secretion levels, resulting in lower circulating levels of IGF-1 (16). Testosterone exerts a stimulatory effect on growth hormone secretion at the pituitary level, which is called a “push effect.” On the other hand, estrogen increases GH secretion only through its inhibitory effect on IGF-1 production in the liver with resultant feedback stimulation of GH release from the pituitary: “a pull effect” (17). Spontaneous and stimulated GH secretion is higher in young women than in postmenopausal women or young men. The difference was found to be strongly correlated with estrogen concentrations (18). GH secretion declines more rapidly with increasing age in men than in women between the third and fifth decades (19). Although spontaneous GH secretion falls progressively with advancing years, an abrupt reduction over the menopausal years does not occur, and most of the change is explained by age-associated increases in the body or abdominal fat (20, 21).

GH circulates in the blood bound to a high-affinity binding protein (GHBP). The liver is a major source of GHBP, which is derived from proteolytic cleavage of the extracellular domain of the GH receptor (GHR). GHBP alters the distribution and pharmacokinetics of GH and is likely to modulate GH action (22). Serum GHBP concentrations are significantly higher in women than in men (23).

There is strong evidence that GH negatively regulates many pro-inflammatory cytokines. Serum levels of CRP, IL-6, and TNF-α are increased in adults with GH deficiency and fall in response to GH replacement (24, 25). Monocyte production of IL-6 and TNF-α is increased in patients with GH deficiency and reduced by GH treatment (24). Levels of CRP are low in active acromegaly and increase with the disease control (26). These data indicate that GH directly or indirectly reduces inflammation by modulating serum levels of cytokines and markers of inflammation. Meanwhile, there is evidence that estrogen inhibits signaling of several members of the cytokine receptor family, including prolactin, IL-6, and leptin (27). Thus, deficiency in both GH and estrogen is associated with increased serum concentrations of CRP, IL-6, and TNF-α. Recent data suggests that the IL-6-JAK-STAT3 axis is closely involved in the development of severe COVID-19 disease (28, 29). High serum IL-6 has been commonly reported in patients with severe COVID-19 and correlated significantly with mortality (30, 31). The inhibitory action of estrogen on JAK2-mediated signal transduction may have broader implications beyond GH action given that the JAK/STAT/SOCS systems are integral components of the cytokine receptor signaling (16). Targeting JAK-STAT signaling to control cytokine release syndrome in COVID-19 has recently been presented as an attractive therapeutic strategy (32).

## Growth Hormone Profile in Potential Risk Factors for Severity of COVID-19 Illness

COVID-19 disease severity and mortality were reported to be increased with some risk factors, such as morbid obesity, hypertension, diabetes, respiratory disorders, excessive alcohol intake, and chronic liver and kidney disease (33–35).

### Morbid Obesity

Morbid obesity occurs when the body mass index (BMI) exceeds 35. It constitutes a risk factor for SARS-CoV-2 severity, which requires increased attention to preventive measures in susceptible individuals (36). In morbid obesity, there is markedly decreased GH secretion. In addition, for both adults and children, the greater the BMI, the lower the GH response to provocative stimuli (37). In obesity, both the spontaneous (38, 39) and stimulated (40) pulsatile patterns of GH secretion controlled by the hypothalamus are blunted. The accumulation of truncal obesity, particularly visceral adipose tissue mass, was found to be a stronger negative determinant of GH secretion than other factors, such as age, sex, or generalized obesity (41, 42). GH deficiency effectively enhances insulin resistance and visceral obesity through increasing cortisol production in key target tissues, including liver and adipose tissue (43). In the peripheral tissues, corticosteroid hormone action is determined, in part, through the activity of 11beta-hydroxysteroid dehydrogenases (11beta-HSD). Two iso-zymes of 11 beta-HSD interconvert hormonally active cortisol (F) and inactive cortisone (E). 11beta-HSD type 1 principally activates F from E in the liver and adipose tissue while 11beta-HSD type 2 inactivates F to E in the kidney and placental tissue. GH acting *via* IGF-1, inhibits 11beta-HSD1, resulting in a shift in cortisol metabolism favoring cortisone production (44). Patients with truncal obesity but with no evidence of hypopituitarism have relative GH deficiency, and it is exciting that low-dose GH treatment in this group, by inhibiting cortisol generation within omental fat, may offer an effective therapeutic approach (43).

### Diabetes Mellitus

Diabetes mellitus is associated with increased severity and mortality of disease in COVID-19 pneumonia (45). Although GH excess, such as in patients with acromegaly, predisposes to diabetes, studies report that there is a significantly increased prevalence of diabetes mellitus in adult GHD patients compared with the general population (46), particularly in those with additional risk factors, such as a family history of diabetes mellitus or obesity (47). The association between GHD and diabetes can be largely explained by adverse body compositions in patients with GHD. The increased abdominal obesity seen in GHD patients is likely a contributor to the decreased insulin sensitivity observed in some patients (48). GH is an important regulator of glucose levels, and adult patients with GHD are reported to have impaired glucose metabolism, insulin resistance, and fasting hyperglycemia (49, 50).

### Hypertension

Hypertension is another reported risk factor for Covid-19 disease. Patients with raised blood pressure were found to have a twofold increased risk of death from COVID-19 compared to normotensive patients (51). In adult GHD, cardiovascular and cerebrovascular morbidity and mortality are increased, and this elevated risk can be largely attributed to hypertension (52). The beneficial effect of GH on cardiovascular risk factors in patients with hypopituitarism may be an indirect effect *via* alteration in cortisol metabolism (43). Previous studies described a close relationship between the GH/IGF-1 axis and the renin-angiotensin-aldosterone axis (RAS). GH has been shown to stimulate RAS as demonstrated by increasing levels of angiotensinogen, aldosterone, and plasma renin activity in human subjects (53). Angiotensin-Converting Enzyme 2 (ACE2), a cell membrane receptor in various target tissues, including the lung, catalyzes angiotensin II conversion to angiotensin-(1–7). The ACE2/angiotensin-(1–7)/MAS axis counteracts the negative effects of the renin-angiotensin system (RAS), thus playing an important role in maintaining the physiological and pathophysiological balance of the body (54). However, the upregulation of the (ACE2)/Angiotensin-(1-7)/Mas receptor axis has been recorded in the heart and the kidney of growth hormone receptor knock-out mice (55). On the other hand, downregulation of the ACE2/angiotensin-(1-7/MAS receptor axis has been recorded in the heart and kidney of transgenic mice overexpressing growth hormone (56). SARS-CoV-2 enters host cells *via* the ACE2 receptor, which is expressed in various human organs, and spike glycoprotein of SARS-CoV-2, which binds to ACE2, represents a potential target for developing specific drugs and vaccines (57). Besides the direct viral effects and inflammatory reactions associated with COVID-19 pathogenesis, ACE2 downregulation that follows COVID-19 infection and the consequent imbalance between the RAS and ACE2/angiotensin-(1–7)/MAS may also contribute to multiple organ injury in COVID-19 (58). Whether adult GHD leads to overexpression of ACE2 *in vivo* is not yet established by clinical studies.

### Respiratory Disease

Meanwhile, in patients with bronchial asthma, it is reported that GH levels and response to GHRH are decreased. Low levels of the hormone are also associated with corticosteroid- and salbutamol-treated asthmatic patients (59).

Also, obese patients with obstructive sleep apnea syndrome (OSAS) demonstrate a peculiar reduction of both spontaneous and stimulated GH secretion coupled with reduced IGF-I levels. These endocrine abnormalities are more marked than those observed in nonapneic obese subjects and are likely to be due to the effects of hypoxia and sleep fragmentation on hormone secretory patterns. The GH/IGF-I axis activity disruption can be responsible, at least in part, for metabolic alterations, which are common in OSAS and increase the risk of cardiovascular events as well as mortality (60). In addition, the serum levels of IGF-1 are significantly reduced in patients with acute exacerbations of COPD (AECOPD) compared with other COPD patients (61) and then increase relatively at the time of recovery. However, the serum levels of IGF-1 both on admission and on the discharge of AECOPD patients are lower than those of healthy subjects (62). Moreover, emphysematous patients seem to have significantly lower IGF-1 levels compared to those with chronic bronchitis both on admission and at discharge (63).

### Chronic Liver Disease

GH resistance is an increasingly recognized feature related to the reduction of IGF-I, IGF-II, and IGFBP-3 in liver dysfunction that may have been further pathogenically affected by the severity of liver dysfunction, the disorder of portosystemic shunting, and malnutrition of hepatic storage. In addition, the production/secretion of GH receptor was also markedly reduced due to severely damaged hepatocytes, thus leading to the disturbance of feedback maladjustment and GH resistance (64, 65). Patients with adult GHD show increased prevalence of nonalcoholic fatty liver disease (NAFLD)/nonalcoholic steatohepatitis (NASH), and GH replacement therapy has been reported to improve these conditions (66). Meanwhile, the IGF-1 and IGFBP-3 levels were shown to be low in cirrhosis patients but to improve after liver transplantation (67).

### Chronic Renal Disease

Chronic renal failure is associated with GH resistance and not GH deficiency (68). Suggested mechanisms of GH resistance in such conditions are reduced density of GH receptors in target tissues as indicated by low GHBP in children and adults with chronic kidney disease (CKD), which is proportionate to the degree of renal dysfunction (69), disturbed GH-activated post-receptor Janus kinase/signal transducer, and activator of transcription (JAK/STAT) signaling (70), and decreased levels of free IGF-1 due to increased inhibitory IGF-binding proteins (IGFBPs) (71). An important regulator of the JAK2/STAT pathway is the suppressor of cytokine signaling (SOCS) proteins, which is induced by GH. These proteins bind to JAK2 and inhibit STAT phosphorylation (72). The upregulation of SOCS has been described in inflammatory conditions and may play a similar role in chronic renal disease (73). The sodium and water-retaining effect of GH has been known for decades (74). Although the exact mechanism behind the antinatriuretic action of GH is not fully clarified, several direct and indirect mechanisms have been suggested. Both GH and IGF-I receptors are expressed in renal tubules (75), suggesting a direct effect of GH and IGF-I on sodium and water retention (76). GH deficiency in children and adults is associated with major changes in body composition, mainly presenting with an increase in subcutaneous and visceral fat mass and low sodium and water body content (77).

### Chronic Alcohol Consumption

Moreover, high alcohol consumption was reported by the WHO to be a high-risk factor for severe Covid-19 illness. This remarkable note raises the possibility of a direct relationship between low consumption of alcohol in certain regions of the world and the limitation of the spread of the disease. The majority of effects of GH appear to be mediated indirectly through the enhanced synthesis and secretion of insulin-like growth factor (IGF)-I. Most studies have shown that chronic alcohol consumption in rodents or humans is associated with a decreased circulating concentration of IGF-I. Meanwhile, excessive alcohol intake has been shown to inhibit spontaneous pulsatile GH secretion (78).

It is, thus, worth noting that growth hormone deficiency and/or resistance could represent an epiphenomenon in potential risk groups for COVID-19 disease. GHD may be implicated in the severity and higher mortality of the disease in those groups of patients.

## The Role of Growth Hormone in Immunity

Studies suggest that endocrine and neuroendocrine systems greatly influence the immune system (79). GH has an important role in the development of the immune system and may enhance the growth of the thymus gland which is responsible for the production of immune cells called T cells, the mediator of cell-mediated immunity (80). GH is also produced by lymphoid organs, such as the thymus, the spleen, and immune cells (81). Besides, clinical studies have suggested a significant role of GH in immune regulation, and the GH receptor is expressed on different subpopulations of lymphocytes (82). GH stimulates T and B cell proliferation and immunoglobulin formation. It enhances the maturation of myeloid progenitor cells and is also able to modulate cytokine response (83). A clinical study in 2012, demonstrated that lower circulating levels of IGF1 were associated with incidence and mortality from adult respiratory distress syndrome (ARDS). These data support the role of the IGF pathway in ARDS (84). Having an immune-regulatory effect in addition to their anabolic effects, growth hormone (GH) and insulin-like growth factor 1 (IGF-1), may act to protect the host from lethal bacterial infection as well. The hormones promote the maturation of myeloid cells, stimulate phagocyte migration, prime phagocytes for the production of superoxide anions and cytokines and enhance the opsonic activity (85).

## Growth Hormone in Critically Ill Patients

Serum GH levels start to increase in the early hours after the onset of critical illness. Both frequency and amplitude of GH pulses are increased, associated with loss of the typical depressions during interpulse periods, which leads to elevated serum concentrations (86). In addition, the hepatic GH receptor function is suppressed. Peripheral GH resistance leads to low circulating levels of IGF-I, IGFBP-3, ALS, and GHBP (87). The combined effect of these alterations in the GH axis leads to a shift from the anabolic effects of IGF-I to more catabolic actions of GH, such as lipolysis, insulin resistance, and immune stimulation (88). When recovery does not start within a week and a chronic phase of critical illness ensues, the pulsatile pattern of GH secretion fades out, and GH peaks become blunted with IGF-I, IGFBP-3, and ALS levels remaining low. Interpulse GH concentrations also decrease but still appear to be higher than in healthy subjects (89). In the acute phase of critical illness, hepatic GH resistance is responsible for altering the GH axis. Whereas, during the prolonged phase of critical illness, the fall in GH is thought to be caused by an impaired hypothalamic drive. Hepatic GH resistance does not appear to persist during chronic critical illness (90). This is supported by a high GH responsiveness to the administration of GH secretagogues (GHRPs) in chronic critically ill patients. In fact, restoration of pulsatile GH secretion pattern associated the administration of GHRP, alone or with the coadministration of GHRH, leading to a sixfold and tenfold increase in amplitudes of GH serum peaks, respectively. Strikingly, the administration of GHRH alone is not capable of restoring the typical pulsatile pattern of GH secretion (91). Another suggested contributor to the attenuated GH levels during chronic critical illness is the scarcity of the active form of ghrelin, the endogenous ligand of the GH secretagogue receptor, and a powerful GH secretagogue (92). The low serum IGF-I and its binding proteins are associated with biochemical markers of impaired anabolism, such as low serum osteocalcin and leptin (93).

A large randomized clinical trial, investigating the effect of high-dose GH injection to prolonged critically ill patients, unexpectedly recorded a doubling in mortality in the intervention cohort (94). Because GH resistance at least partially resolves in the chronic phase, it is likely that such high doses of GH and, consequently, high levels of IGF-I, evoke toxic side effects, such as excessive fluid retention, hypercalcemia, and pronounced insulin resistance with hyperglycemia. Although small studies show the ability of GHRP-2 to restore a normal GH pulsatile pattern in severely ill patients, and of the combination of GHRP-2 and thyrotropin-releasing hormone (TRH) to induce an anabolism and suppress catabolism in prolonged critically ill patients (95), the clinical outcome of infusion with GH secretagogues has not yet been studied. Also, substitution with ghrelin has recently been investigated in smaller animals and *in vitro* studies and appeared to enhance autophagy, reduce catabolism, and improve hemodynamics (96). As ghrelin induces appetite, infusion of ghrelin during the chronic phase of critical illness when patients restart oral intake may enhance food intake and could lead to an improvement in clinical outcomes (97). Large-scale RCTs in humans, to corroborate these findings, have not yet been performed.

## Growth Hormone and Cancer Risk

The use of GH, especially in non-GHD persons, has raised safety concerns regarding the cancer risk because IGF-I has potent mitogenic and antiapoptotic effects. However, the results of epidemiological, experimental, and observational studies are ambiguous. A number of biases and confounders affect the interpretation of data (98). Nevertheless, the concern of cancer risk associating administration of r-HGH is related to long-term therapy, especially in children with GHD and in particular, those who recovered from malignancy for fear of recurrence.

## Adverse Effects of GH Treatment

Older persons are more sensitive to replacement with GH and more susceptible to the side effects of therapy. The acute side effects are due to the hormonal effects of over-replacement, which can be avoided or relieved with careful dose titration. Patients who are older, heavier, or female are more prone to develop complications (99). Common side effects of GH replacement include fluid retention, peripheral edema, arthralgia, and carpal tunnel syndrome. Although glucose levels often increase with the initiation of GH, these levels generally return toward normal with the improvement in body composition and reduced insulin resistance. Other, less frequently reported side effects are headache, tinnitus, and benign intracranial hypertension (99). Whether increasing growth hormone above the age-appropriate normal range may have as many risks, both acute and delayed, as benefits is a worthwhile hypothesis to examine.

## Conclusion

In conclusion, we have observed some characteristic distribution of the severity of the disease COVID-19 that seems to be matched with a relative deficiency and/or resistance of growth hormone in some groups of patients. We wanted to share that note with the medical community so that it may add to the urgent efforts to understand and consequently overcome the pandemic. We think that there is a need for further studying the role of growth hormone in the unique epidemiological pattern of COVID-19 so that it can help in the early detection and management of the high-risk groups. A randomized controlled trial would help clarify the possible prophylactic role of growth hormone supplement in those groups of patients to alleviate immunity and decrease the severity and/or mortality of the disease till an efficient vaccine is available in the market worldwide.

## Data Availability Statement

The original contributions presented in the study are included in the article/supplementary material. Further inquiries can be directed to the corresponding author.

## Author Contributions

All authors listed have made a substantial, direct, and intellectual contribution to the work and approved it for publication.

## Conflict of Interest

The authors declare that the research was conducted in the absence of any commercial or financial relationships that could be construed as a potential conflict of interest.
